# The phylogenetic relationships and species richness of host-specific *Dactylogyrus* parasites shaped by the biogeography of Balkan cyprinids

**DOI:** 10.1038/s41598-018-31382-w

**Published:** 2018-08-29

**Authors:** Michal Benovics, Yves Desdevises, Jasna Vukić, Radek Šanda, Andrea Šimková

**Affiliations:** 1 Department of Botany and Zoology, Faculty of Science, Masaryk University, Kotlářská 2, 61137 Brno, Czech Republic; 2 Sorbonne Universités, UPMC Univ Paris 06, CNRS, Biologie Intégrative des Organismes Marins (BIOM), Observatoire Océanologique de Banyuls/Mer, F-66650 Banyuls/Mer, France; 3 Department of Ecology, Faculty of Science, Charles University in Prague, Viničná 7, 128 44 Prague, Czech Republic; 4 National Museum, Václavské Náměstí 68, 115 79 Prague, Czech Republic

## Abstract

Parasites exhibiting a high degree of host specificity are expected to be intimately associated with their hosts. Therefore, the evolution of host-specific parasites is at least partially shaped by the evolutionary history and distribution of such hosts. Gill ectoparasites of *Dactylogyrus* (Monogenea) are specific to cyprinid fish. In the present study, we investigated the evolutionary history of 47 *Dactylogyrus* species from the Balkan Peninsula, the Mediteranean region exhibiting the highest cyprinid diversity in Europe, and from central European cyprinids. Phylogenetic analyses revealed four well-supported clades of endemic and non-endemic *Dactylogyrus* spp. with four basal taxa. Endemic cyprinids with a limited distribution range were parasitized by endemic *Dactylogyrus* species, but some of them shared several *Dactylogyrus* species with central European cyprinids. Species delimitation analyses based on molecular data suggest that *Dactylogyrus* diversity is higher than that defined from morphology. Some endemic cyprinid species harboured *Dactylogyrus* species of different origins, this probably resulting from multiple host switching. Our results support the view that the evolution of *Dactylogyrus* in the Balkans has been influenced not only by the historical dispersion and distribution of their cyprinid hosts, but also by recent contacts of non-native cyprinid species with endemic cyprinid fauna in this region.

## Introduction

The species richness of parasitic taxa and their distribution in host species is usually closely related to the history, dispersion and diversity of their hosts^1–3^. The parasitic genus *Dactylogyrus* (Monogenea), known for its wide species richness (over 900 nominal species according to Gibson *et al*.^4^), is restricted mainly to fish species of Cyprinidae, a highly diversified group of primarily freshwater fish^5^. *Dactylogyrus* species exhibit a high degree of host specificity within the multitude of their host species^6^.

Previous studies suggest that each cyprinid species can host at least one *Dactylogyrus* species^7–9^. Within one host species the distribution of *Dactylogyrus* species is restricted to specific microhabitats, i.e. different *Dactylogyrus* species occupy distinct niches within host gills^10–12^. The evolution of niche preference is linked with changes of at least one parameter determining niche position on fish gills (e.g. the changes in the positions among the different gill arches or different segments of a given gill arch)^6^. It has been hypothesized that, over evolutionary time, monogeneans developed copulatory organs of different shapes and sizes, which resulted in reproductive isolation within overlapping microhabitats^13^. This was previously documented in *Dactylogyrus* species as well^14^.

Unlike central and northern Europe, where the cyprinid fauna is relatively uniform, southern European peninsulas are extremely rich in endemic cyprinid species^15^. The endemic cyprinid fauna of Mediterranean regions consists of several highly diversified genera whose origin and historical biogeography are still poorly known in spite of several recent studies^16–20^. Zardoya *et al*.^21^ investigated 15 lineages (52 species) of Greek cyprinids and proposed that species related to Danubian cyprinid fauna colonized the Balkan Peninsula during two different time periods. The first one occurred during the Miocene, when fish species such are *Barbus cyclolepis*^22^*, Alburnoides strymonicus*^19^*, Telestes beoticus*, *T. pleurobipunctatus*^20^, and *Squalius peloponensis*^18^ diverged. These species show relatively high molecular divergence in comparison to central European sister group taxa. The second period is related to the Plio-Pleistocene connection of the Balkan Peninsula and the River Danube via river captures^23,24^. This dispersion event included species such are *Barbus balcanicus*^25^, *Squalius vardarensis* and species of *Chondrostoma* and *Alburnus* genera^26^, which exhibit a much lower degree of molecular divergence with respect to Danubian-related taxa. Previous studies on the phylogeny of Balkan cyprinids are focused on *Squalius*^18,26–30^, which is one of two genera (with *Barbus*) inhabiting all three southern European peninsulas. According to the above-cited study by Sanjur *et al*.^30^, based on analysis of the mitochondrial cytochrome *b* gene, Balkan *Squalius* species are grouped into three major clades. Several studies, based on different molecular markers and the analysis of several morphological traits, suggested that the Balkan *Squalius* species with the greatest ancestral diversification is *Squalius keadicus*, which split from other *Squalius* lineages approximately 9 Mya^24,26^. The Balkan ancient lake system, known as Dessaretes, emerged in the Pliocene, and was suggested to have play an important role in freshwater biota speciation processes. For this reason, it is considered to have been a hotspot of endemic Balkan biodiversity^31–35^. The Dessaretes lake system formerly included Lake Ohrid (located in Albania and F.Y.R.O.M.), Lake Prespa (Albania, Greece, F.Y.R.O.M.), Lake Mikri Prespa (Albania, Greece) and Lake Maliq (Albania). Recently, the current distribution of many cyprinid species from the “Dessaretes” region was reevaluated. For example, *Barbus prespensis*, initially known as an endemic species from Lake Prespa, was recently shown to be widespread in the south-eastern Adriatic basin, together with other presumably endemic species from Lake Prespa, namely *Alburnoides prespensis* and *Squalius prespensis*^19,25,36^. This basin is a part of the evaporated Lake Maliq, historically connected to Lake Prespa and drained after the Second World War^33^.

Gregory^37^ suggested that hosts with a larger area of distribution are infected by more parasitic species. Concerning cyprinids, widely distributed species across Europe such as *Rutilus rutilus* and *Squalius cephalus* harbour up to 9 *Dactylogyrus* species^11,38^. In contrast, Dupont and Lambert^7^ found only 5 *Dactylogyrus* species on *Rutilus rubilio*, an endemic cyprinid species in the Apennine Peninsula. A phylogenetic reconstruction including 51 *Dactylogyrus* species and based on molecular data suggested that species parasitizing central European cyprinids form three monophyletic groups^11^ and are associated with different phylogenetic lineages of cyprinid species representing subfamilies with different origins, histories, and biogeographical distributions. Since studies of endemic and non-endemic *Dactylogyrus* from Balkan cyprinids are scarce and mainly based on morphological data^7,39–41^, the evolutionary histories and patterns of endemism of these host-specific species are still unresolved. Several previous studies concerning different regions of the northern Mediterranean Sea suggested that endemic cyprinids harbour endemic *Dactylogyrus* species^7,9,42^. Some phylogenetic studies were focused on *Dactylogyrus* species from selected cyprinid genera, such as *Dactylogyrus* spp. parasitizing *Barbus* species^43^. According to the authors, such *Dactylogyrus* species are supposed to exhibit both genetic and morphological variabilities between different host species. Dupont^44^ investigated the historical biogeography of *Dactylogyrus* species of endemic *Rutilus, Luciobarbus*, and *Pachychilon* hosts from the Balkan Peninsula and suggested that the endemism of *Dactylogyrus* can be explained by the formation of landmass and freshwater streams during the Neogene and Pleistocene eras.

The aim of the present study was to investigate the diversity, evolutionary history, and phylogenetic relationships of *Dactylogyrus* spp. parasitizing endemic cyprinids of the Balkan Peninsula. First, we analyzed the degree of endemism in *Dactylogyrus* species parasitizing these cyprinids. Next, we focused on the phylogenetic relationships between endemic *Dactylogyrus* and commonly distributed *Dactylogyrus* (species shared between central European and endemic Balkan cyprinid species) in order to infer potential scenarios of historical contact between different cyprinids. Concerning *Dactylogyrus* species with a wide host range, we also searched for genetic structuration by analyzing the level of genetic diversity and its correlation with the geographical distances between their hosts. Finally, we assessed the species status of generalist *Dactylogyrus* on the basis of molecular data in order to test whether the degree of genetic variability was in concordance with the current species status based on a classical taxonomical approach.

## Results

### Dactylogyrus species richness

A total of 53 *Dactylogyrus* species were identified from cyprinid hosts from the Balkans (Table 1) and central Europe. 47 species were collected from endemic Balkan cyprinids. Six additional species were collected from the Czech Republic and included in analyses. Balkan cyprinids were parasitized by 1 to 5 *Dactylogyrus* species with an average of 2 species per host species. The highest *Dactylogyrus* species diversity was reported on representatives of the genera *Pachychilon – P. pictum* (5); *Squalius – S. squalus* (4) and *S. prespensis* (4); *Barbus – B. prespensis* (4); and *Rutilus – R. basak* (4), *R. lacustris* (4), and *R. ohridanus* (4). Eight *Dactylogyrus* species were unidentified and are expected to be new to science. These potentially new species were collected from the following host species: *Delminichthys adspersus*, *Chondrostoma knerii*, *Squalius tenellus, Luciobarbus albanicus, L. graecus, Tropidophoxinellus spartiaticus, Telestes karsticus* and *Pachychilon macedonicum*.

**Table 1:** List of collected *Dactylogyrus* species and their cyprinid host species.

*Dactylogyrus* species	Host	Locality	partial 18S + ITS1	partial 28S
*D. auriculatus*	*Abramis brama*	CZ1	MG792838*	MG792952*
*D. alatus*	*Alburnus neretvae*	B1	MG792842*	MG792956*
	*Alburnus neretvae*	B2	MG792843*	MG792957*
*D. anchoratus*	*Carassius gibelio*	C2	KY859795	KY863555
*D. balkanicus*	*Barbus plebejus*	C1	MG792861*	MG792976*
	*Barbus prespensis*	G1	KY201093	KY201107
	*Barbus rebeli*	A6	MG795863*	MG792978*
*D. borealis*	*Phoxinus* sp.	B9	KY629343	KY629372
*D. caballeroi*	*Rutilus ohridanus*	A4	MG792902*	MG793018*
	*Rutilus rutilus*	CZ1	AJ564114	MG793022*
*D. carpathicus*	*Barbus barbus*	CZ1	KY201098	KY201111
*D. caucasicus*	*Alburnoides devoli*	A1	MG792840*	MG792954*
	*Alburnoides fangfangae*	A2	MG792841*	MG792955*
	*Alburnoides prespensis*	G1	MG792847*	MG792961*
*D. cornu*	*Vimba vimba*	CZ1	KY629342	KY629371
*D. crivellius*	*Barbus balcanicus*	G4	MG792854*	MG792969*
	*Barbus peloponnesius*	G7	KY629339	KY629368
	*Barbus plebejus*	C1	MG792862*	MG792977*
	*Barbus prespensis*	G1	KY201094	KY201108
	*Barbus rebeli*	A6	MG792863*	MG792979*
	*Barbus* sp.	A7	MG792866*	MG792981*
*D. crucifer*	*Rutilus lacustris*	G12	MG792898*	MG793014*
	*Rutilus rutilus*	CZ1	AJ564120	KY629374
*D. difformis*	*Scardinius plotizza*	B4	MG792908*	MG793025*
*D. difformoides*	*Scardinius plotizza*	B4	MG792909*	MG793026*
*D. dirigerus*	*Chondrostoma ohridana*	G1	MG792873*	MG792988*
	*Chondrostoma vardarensis*	G2	MG792876*	MG792991*
	*Chondrostoma vardarensis*	G3	MG792877*	MG792992*
*D. dyki*	*Barbus balcanicus*	G4	MG792855*	MG792970*
	*Barbus barbus*	CZ1	KY629338	KY629367
	*Barbus cyclolepis*	G5	MG792856*	MG792971*
	*Barbus peloponnesius*	G6	MG792858*	MG792973*
	*Barbus peloponnesius*	G7	MG792859*	MG792974*
	*Barbus prespensis*	A5	KY201095	KY201109
	*Barbus prespensis*	G1	KY859804	KY859803
	*Barbus rebeli*	A6	MG792865*	MG792980*
	*Barbus sperchiensis*	G8	MG792867*	MG792982*
	*Barbus strumicae*	G1	MG792868*	MG792983*
*D. ergensi*	*Chondrostoma knerii*	B4	MG792870*	MG792985*
	*Chondrostoma ohridana*	G1	MG792874*	MG792989*
	*Chondrostoma vardarensis*	G2	MG792878*	MG792993*
*D. erhardovae*	*Rutilus aula*	C2	MG792893*	MG793009*
	*Rutilus basak*	B10	MG792894*	MG793010*
*D. extensus*	*Cyprinus carpio*	—	KM277459	AY553629
*D. fallax*	*Chondrostoma nasus*	CZ1	MG792872*	MG792987*
	*Rutilus rutilus*	CZ1	MG792906*	MG793023*
	*Vimba vimba*	CZ1	KY629341	KY629370
*D. folkmanovae*	*Squalius cephalus*	CZ1	MG792912*	MG793029*
	*Squalius cephalus*	B7	MG792911*	MG793028*
	*Squalius orpheus*	G9	MG792916*	MG793035*
	*Squalius platyceps*	A8	MG792919*	MG793038*
	*Squalius prespensis*	A9	MG792921*	MG793040*
	*Squalius prespensis*	G1	MG792922*	MG793041*
	*Squalius* sp.	G10	MG792926*	MG793032*
	*Squalius squalus*	C4	MG792928*	MG793044*
	*Squalius vardarensis*	G4	MG792935*	MG793049*
*D. formosus*	*Carassius gibelio*	C2	MG792869*	MG792984*
*D. ivanovichi*	*Pachychilon pictum*	G1	MG792883*	MG792999*
*D. izjumovae*	*Scardinius dergle*	C1	MG792907*	MG793024*
	*Scardinius plotizza*	B4	MG792910*	MG793027*
*D. malleus*	*Barbus barbus*	CZ1	KY201099	KY201112
*D. martinovici*	*Pachychilon pictum*	A8	MG792884*	MG793000*
	*Pachychilon pictum*	G1	MG792885*	MG793001*
*D. minor*	*Alburnus scoranza*	A4	MG792848*	MG792962*
*D. nanoides*	*Squalius cephalus*	B7	MG792913*	MG793030*
	*Squalius prespensis*	G1	MG792923*	MG793045*
	*Squalius squalus*	B11	MG792929*	MG793046*
*D. omenti*	*Aulopyge huegelii*	B3	KY201091	KY201105
*D. parvus*	*Alburnus scoranza*	A4	MG792849*	MG792963*
*D. petenyi*	*Barbus balcanicus*	G4	KY201097	KY201113
	*Barbus cyclolepis*	G5	MG792857*	MG792972*
	*Barbus peloponnesius*	G7	MG792860*	MG792975*
*D. petkovici*	*Pachychilon pictum*	A8	MG792886*	MG793002*
	*Pachychilon pictum*	G1	MG792887*	MG793003*
*D. prespensis*	*Barbus prespensis*	G1	KY201096	KY201110
*D. prostae*	*Squalius cephalus*	CZ1	MG792914*	MG793031*
	*Squalius pamvoticus*	G13	MG792917*	MG793036*
	*Squalius prespensis*	G1	MG792924*	MG793042*
	*Squalius* sp.	G10	MG792927*	MG793033*
*D. rarissimus*	*Alburnus neretvae*	B1	MG792844*	MG792958*
	*Alburnus neretvae*	B2	MG792845*	MG792959*
	*Pelasgus laconicus*	G11	MG792890*	MG793006*
	*Rutilus basak*	B10	MG792895*	MG793011*
	*Rutilus lacustris*	G12	MG792899*	MG793015*
	*Rutilus ohridanus*	A4	MG792903*	MG793019*
	*Telestes alfiensis*	G15	MG792938*	MG793055*
	*Telestes dabar*	B12	MG792939*	MG793056*
	*Telestes fontinalis*	C6	MG792940*	MG792997*
	*Telestes metohiensis*	B13	MG792944*	MG793059*
*D. rosickyi*	*Pachychilon pictum*	G1	MG792888*	MG793004*
*D. rutili*	*Rutilus basak*	B10	MG792896*	MG793012*
	*Rutilus lacustris*	G12	MG792900*	MG793016*
	*Rutilus ohridanus*	A4	MG792904*	MG793020*
*D. rysavyi*	*Alburnoides thessalicus*	G3	MG792851*	MG792965*
*D. sekulovici*	*Pachychilon pictum*	G1	MG792889*	MG793005*
*D. soufii*	*Telestes montenigrinus*	A10	MG792946*	MG793061*
*Dactylogyrus* sp. 1	*Squalius tenellus*	B5	MG792933*	MG793050*
*Dactylogyrus* sp. 2	*Luciobarbus graecus*	G8	KY201101	KY201115
*Dactylogyrus* sp. 3	*Luciobarbus albanicus*	G10	KY201100	KY201114
*Dactylogyrus* sp. 4	*Delminichthys adspersus*	B6	MG792881*	MG792995*
*Dactylogyrus* sp. 5	*Pachychilon macedonicum*	G3	MG792882*	MG792998*
*Dactylogyrus* sp. 6	*Tropidophoxinellus spartiaticus*	G6	MG792950*	MG793065*
*Dactylogyrus* sp. 7	*Chondrostoma knerii*	B4	MG792871*	MG792986*
*Dactylogyrus* sp. 8	*Telestes karsticus*	C7	MG792942*	MG793057*
*D. sphyrna*	*Rutilus basak*	B10	MG792897*	MG793013*
	*Rutilus ohridanus*	A4	MG792905*	MG793021*
	*Vimba vimba*	CZ1	MG792951*	MG793066*
*D. suecicus*	*Rutilus lacustris*	G12	MG792901*	MG793017*
	*Telestes montenigrinus*	A10	MG792947*	MG793062*
*D. tissensis*	*Alburnoides thessalicus*	G3	MG792852*	MG792966*
*D. vastator*	*Aulopyge huegelii*	B3	KY201092	KY201106
	*Carassius gibelio*	CZ2	KY201103	KY629366
*D. vistulae*	*Alburnoides ohridanus*	A3	MG792846*	MG792960*
	*Alburnoides strymonicus*	G2	MG792850*	MG792964*
	*Alburnoides thessalicus*	G3	MG792853*	MG792968*
	*Chondrostoma ohridana*	G1	MG792875*	MG792990*
	*Chondrostoma phoxinus*	B5	MG792880*	MG792994*
	*Chondrostoma vardarensis*	G3	MG792879*	MG792967*
	*Phoxinellus alepidotus*	B7	MG792891*	MG793007*
	*Phoxinellus pseudalepidotus*	B8	MG792892*	MG793008*
	*Squalius illyricus*	C3	MG792915*	MG793034*
	*Squalius peloponensis*	G14	MG792918*	MG793037*
	*Squalius platyceps*	A8	MG792920*	MG793039*
	*Squalius prespensis*	A9	KY629340	KY629369
	*Squalius prespensis*	G1	MG792925*	MG793043*
	*Squalius squalus*	B11	MG792930*	MG793047*
	*Squalius svallize*	C5	MG792932*	MG793049*
	*Squalius tenellus*	B5	MG792934*	MG793051*
	*Squalius vardarensis*	G4	MG792936*	MG793053*
	*Telestes fontinalis*	C6	MG792941*	MG792996*
	*Telestes karsticus*	C7	MG792943*	MG793058*
	*Telestes metohiensis*	B13	MG792945*	MG793060*
	*Telestes montenigrinus*	A10	MG792948*	MG793063*
	*Telestes pleurobipunctatus*	G7	MG792949*	MG793064*
*D. vranoviensis*	*Squalius squalus*	B11	MG792931*	MG793048*
	*Squalius vardarensis*	G4	MG792937*	MG793054*
*D. zandti*	*Abramis brama*	CZ1	MG792839*	MG792953*

GenBank accession numbers are included. New sequences obtained in this study are marked by asterisks (*).

### Phylogenetic analyses and genetic distances

**Figure 1:**
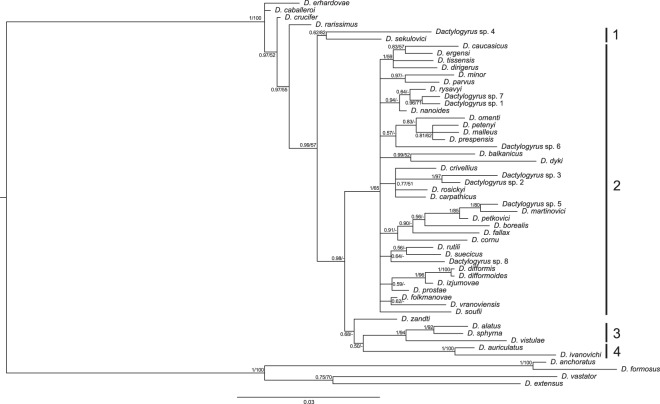
*Phylogram of 54 Dactylogyrus species from the Balkans and Central Europe reconstructed by Bayesian inference*. The tree is based on concatenated data of partial 18S rDNA and partial 28S rDNA sequences. Values along branches indicate posterior probabilities and boostrap values resulting from Bayesian inference and Maximum likelihood analyses, respectively. Values <0.80 for BI and <50% for ML are indicated by dashes (-). Branch lengths correspond to the expected number of substitutions per site. Labels 1–4 refer to different *Dactylogyrus* lineages. The phylogenetic tree was rooted using *Dactylogyrus* species parasitising *Carassius gibelio* and *Cyprinus carpio* (following Šimková *et al*.^12^).

The concatenated sequence alignment of partial 18S and partial 28S rDNA from representatives of 54 *Dactylogyrus* species from the Balkan Peninsula and central Europe contained 1158 unambiguous nucleotide positions. The data were treated as partitioned and GTR+I was selected as the most optimal evolutionary model for the 446 bp-long partial 18S rDNA sequences, and GTR+I+G for the 712 bp-long partial 28S rDNA sequences. BI (Bayesian inference) and ML (Maximum Likelihood) analyses produced trees with identical topologies which varied in node support values (Fig. 1). The resulting phylogram divided most of the species into 4 strongly-to-moderately supported clades. Four *Dactylogyrus* species (*D. erhardovae*, *D. caballeroi*, *D. crucifer* and *D. rarissimus*) were placed in an external position to these four clades. The first clade (clade 1), weakly supported by BI and well supported by ML analyses, included the species *D. sekulovici* from *Pachychilon pictum* and *Dactylogyrus* sp. 4 from *Delminichthys adspersus*. The second clade (clade 2), highly supported by BI and weakly supported by ML analyses, was the largest and included all species parasitizing *Barbus* and *Luciobarbus*. *Dactylogyrus* species endemic for the Balkan Peninsula and also widely distributed *Dactylogyrus* species clustered in this second clade. Generally, species with similarly shaped haptoral hard parts clustered together and such clusters were well or moderately supported by at least BI analysis (PP, posterior probability > 0.81). For example, *D. petkovici*, *D. martinovici* and *Dactylogyrus* sp. 5, representing a monophyletic group, share a similar type of thin anchor hooks and a ventral bar with five extremities, while *Dactylogyrus* sp. 2 and *Dactylogyrus* sp. 3, representing another monophyletic group, display hard parts of the haptor that are almost indistinguishable in shape. Three *Dactylogyrus* species from *Barbus* (i.e. *D. petenyi*, *D. malleus* and *D. prespensis*, which also share a similar shape of their haptoral hard parts) were clustered with *D. omenti* from *Aulopyge huegelii*. The third clade was strongly supported by both BI and ML analyses and included *D. alatus*, *D. sphyrna* and *D. vistulae*, which are large worms with large haptoral anchor hooks. The last well-supported clade (PP = 1, BS, bootstrap value = 100) included *D. auriculatus* from *Abramis brama* and *D. ivanovichi* from *P. pictum* (clade 4), which exhibited identically shaped MCO (male copulatory organ) hard parts but VA (vaginal armament) of slightly different shape. All species from clades 3 and 4, except *D. alatus*, had no connective ventral bar. *Dactylogyrus zandti* appeared to be a sister species to clades 3 and 4, but its position was not supported.

**Figure 2:**
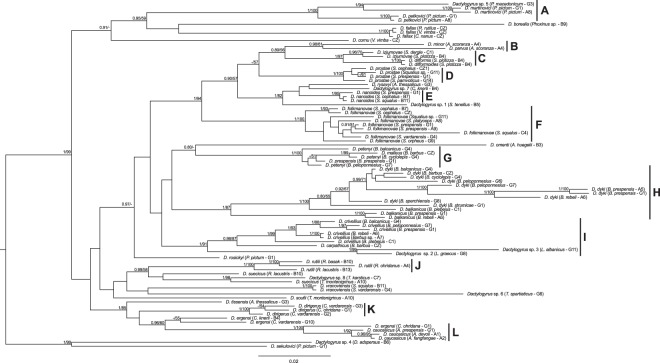
*Phylogram of selected Dactylogyrus species from the Balkans and Central Europe constructed by Bayesian inference*. The tree is based on concatenated data of partial 18S rDNA, ITS1 region and partial 28S rDNA sequences. Values along branches indicate posterior probabilities and boostrap values resulting from Bayesian inference and maximum likelihood analyses, respectively. Values <0.80 for BI and <50% for ML are indicated by dashes (-). Branch lengths correspond to number of substitutions per site. Labels A–L refer to different, well supported, *Dactylogyrus* clades.

To resolve the phylogenetic relationships among groups within the second clade, we used a concatenated alignment of partial 18S, 28S rDNA, and the highly variable ITS1 (Internal Transcribe Spacer 1) region. The alignment of 86 sequences comprised 1503 unambiguously aligned nucleotide positions.The most optimal evolutionary models were TrNef+I for the alignment of 446 bp-long partial 18S rDNA sequences, SYM+G for the alignment of 344 bp-long ITS1 sequences, and TVMef+I+G for the alignment of 713 bp-long partial 28S rDNA sequences. BI and ML analyses generated trees with the same topologies (Fig. 2). The resulting trees were rooted using clade 1 from the first phylogenetic reconstruction (Fig. 1).

The phylogenetic analyses divided clade 2 into several strongly-to-moderately supported groups. Group A included species parasitizing *Pachychilon*, these sharing the same type of haptoral ventral bar with five radii, similar to the ‘cornu’ type^45^. This monophyletic group of *Dactylogyrus* spp. from *Pachychilon* was highly supported by both BI and ML analyses. All *Dactylogyrus* species of *Scardinius* (*D. difformis, D. difformoides* and *D. izjumovae*) formed a highly supported monophyletic group (group C). The group of two *Dactylogyrus* species from *Alburnus* (group B) formed a sister clade to the abovementioned species from *Scardinius*. *Dactylogyrus prostae, D. nanoides*, and *D. folkmanovae* from *Squalius* formed three very strongly supported monophyletic groups (groups D, E, and F, respectively). Group E also clustered with *D. rysavyi* from *A. thessalicus*, *Dactylogyrus* sp. 7 from *C. knerii*, and *Dactylogyrus* sp. 1 from *S. tenellus*, with strong support from both analyses. All three species exhibit a similarly shaped MCO and parasitize phylogenetically closely related cyprinid lineages^26,45^.

The phylogenetic relationships between *Dactylogyrus* spp. of *Barbus* and those of *Luciobarbus* were unresolved. However, *Dactylogyrus* spp. of these cyprinids formed three well supported groups (G, H and I). All specimens of *D. crivellius*, collected from six *Barbus* species in the Balkans, formed a strongly supported clade. This species clustered with *D. carpathicus* from *B. barbus*. The group of *D. crivellius* and *D. carpathicus* was sister to the group including two *Dactylogyrus* species (sp. 2 and sp. 3) of Balkan *Luciobarbus* spp. (within group I). While *Dactylogyrus* sp. 2 and *Dactylogyrus* sp. 3 were found to be almost identical on the basis of morphological characters, they differed at the molecular level (concatenated partial 18S rDNA and ITS1 region, *p*-distance = 0.041). Our results did not support the monophyly of *D. petenyi*, as this species clustered with *D. malleus* and *D. prespensis* (group G). *Dactylogyrus omenti* from *Aulopyge huegelii* appears also to be phylogenetically closely related to the species parasitizing *Barbus* and *Luciobarbus*, but its position was only moderately supported by BI analysis. The position of *D. rosickyi* of *P. pictum* was also uncertain; however, BI analysis strongly supported its position within the clade including groups C–I. *Dactylogyrus rutili* from *Rutilus* formed a well-supported group (group J) and, according to our results, appears to be phylogenetically closely related to *D. suecicus* (whose monophyly was not supported) and *Dactylogyrus* sp. 8 from *T. karsticus*. Surprisingly, *D. ergensi* collected from three host species formed a paraphyletic group. *Dactylogyrus ergensi* from *C. ohridana* was phylogenetically related to *D. caucasicus*, parasitizing on *Alburnoides* species (group L), in contrast to other *D. ergensi* specimens collected from *C. knerii* and *C. vardarensis*. Nonetheless, *D. caucasicus, D. dirigerus* and *D. ergensi* (included in groups K and L) share a similarly shaped MCO.

The computation of genetic distances between specimens of generalist *Dactylogyrus* species revealed moderate-to-high interpopulation genetic variability. Pairwise genetic distances were calculated for *D. vistulae, D. rarissimus*, and *D. folkmanovae* after eliminating all positions containing gaps and missing data. The selected species are representatives of *Dactylogyrus* with a wide distribution range in Europe. While *D. folkmanovae* is a parasite only of *Squalius* spp., *D. vistulae* and *D. rarissimus* are real generalists parasitizing on species of different cyprinid genera. An alignment of 994 nucleotide positions was used for *D. vistulae* collected from 24 cyprinid species of six genera at 20 localities across the Balkan Peninsula and the Czech Republic. Pairwise sequence diversities varied from 0.000 to 0.020 (Table 2). Generally, geographically adjacent populations were more similar at the molecular level, a finding supported by the Mantel test (P = 0.015). *Dactylogyrus vistulae* from *S. tenellus, S. svallize, S. illyricus, Phoxinellus pseudalepidotus, P. alepidotus*, and *T. metohiensis* were genetically identical and all their host species were from the Dalmatian ichthyogeographical district. The same pattern was observed for *D. vistulae* specimens from *C. nasus* and *Leuciscus idus*, both from central Europe: they were similar at the molecular level. One of the few exceptions was *D. vistulae* from *S. cephalus* in the Czech Republic, which was genetically more similar to Balkan populations collected from *S. squalus* and *S. vardarensis* than to central European populations. *Dactylogyrus rarissimus* was collected from 11 species including four cyprinid genera – *Alburnus, Pelasgus, Rutilus* and *Telestes*. After removing gaps and missing data, the final alignment contained a total of 978 nucleotide positions. The interpopulation genetic variability ranged from 0.001 to 0.030 (Table 3). The pairwise distances revealed that *D. rarissimus* from *R. rutilus* and *R. lacustris* were the most similar (*p*-distance = 0.003). Specimens of *D. rarissimus* from *T. alfiensis* were the most genetically dissimilar to all other specimens collected from other host species (*p*-distance > 0.021). Regarding *D. rarissimus*, the Mantel test did not reveal any significant spatial genetic structure (P > 0.05). *Dactylogyrus folkmanovae* specimens were collected from seven *Squalius* species at nine localities from the Balkans and central Europe. The final alignment contained 977 positions and genetic distances varied from 0.002 to 0.037 (Table 4). Interpopulation genetic variability was found even between specimens collected from two populations of one host species, namely *S. prespensis* (*p*-distance = 0.002), where both populations were in the same ichthyogeographical district. Surprisingly, the same genetic distance was observed between *D. folkmanovae* specimens collected from *S. cephalus* in Bosnia and Herzegovina and from *S. cephalus* in the Czech Republic. The Mantel test indicated a positive correlation between genetic and geographical distance for *D. folkmanovae* populations (P = 0.001).

**Table 2:** Uncorrected pairwise genetic distances between individuals of *D. vistulae* collected from different host species.

No.	Species	LocID	Accession number	1	2	3	4	5	6	7	8	9	10	11	12	13	14	15	16	17	18	19	20	21	22	23	24
1	*Alburnoides ohridanus*	A3	MG792846																								
2	*Alburnoides strymonicus*	G2	MG792850	0.008																							
3	*Alburnoides thessalicus*	G3	MG795853	0.003	0.007																						
4	*Chondrostoma nasus*	CZ1	AJ564160	0.013	0.015	0.012																					
5	*Chondrostoma ohridana*	G1	MG792875	0.007	0.011	0.008	0.014																				
6	*Chondrostoma vardarensis*	G3	MG792879	0.014	0.016	0.012	0.010	0.015																			
7	*Chondrostoma phoxinus*	B5	MG792880	0.008	0.012	0.009	0.019	0.013	0.020																		
8	*Leuciscus idus*	CZ	AJ564162	0.011	0.013	0.010	0.002	0.012	0.008	0.017																	
9	*Phoxin ellus alepidotus*	B7	MG792891	0.007	0.011	0.008	0.018	0.012	0.019	0.001	0.016																
10	*Phoxinellus pseudale-pidotus*	B8	MG792892	0.007	0.011	0.008	0.018	0.012	0.019	0.001	0.016	—															
11	*Squalius cephalus*	CZ1	AJ564161	0.001	0.007	0.002	0.012	0.006	0.013	0.007	0.010	0.006	0.006														
12	*Squalius illyricus*	C3	MG792915	0.007	0.011	0.008	0.018	0.012	0.019	0.001	0.016	—	—	0.006													
13	*Squalius peloponensis*	G14	MG792918	0.006	0.010	0.007	0.015	0.011	0.018	0.010	0.013	0.009	0.009	0.005	0.009												
14	*Squalius platyceps*	A8	MG792920	0.004	0.008	0.005	0.013	0.009	0.016	0.008	0.011	0.007	0.007	0.003	0.007	0.004											
15	*Squalius prespensis*	A9	KY629340	0.003	0.007	0.004	0.014	0.008	0.015	0.007	0.012	0.006	0.006	0.002	0.006	0.005	0.001										
16	*Squalius prespensis*	G1	MG792925	0.003	0.007	0.004	0.014	0.008	0.015	0.007	0.012	0.006	0.006	0.002	0.006	0.005	0.001	—									
17	*Squalius squalus*	B11	MG792930	0.001	0.007	0.002	0.012	0.006	0.013	0.007	0.010	0.006	0.006	—	0.006	0.005	0.003	0.002	0.002								
18	*Squalius svallize*	C5	MG792932	0.007	0.011	0.008	0.018	0.012	0.019	0.001	0.016	—	—	0.006	—	0.009	0.007	0.006	0.006	0.006							
19	*Squalius tenellus*	B5	MG792934	0.007	0.011	0.008	0.018	0.012	0.019	0.001	0.016	—	—	0.006	—	0.009	0.007	0.006	0.006	0.006	—						
20	*Squalius vardarensis*	G4	MG792936	0.001	0.007	0.002	0.012	0.006	0.013	0.007	0.010	0.006	0.006	—	0.006	0.005	0.003	0.002	0.002	—	0.006	0.006					
21	*Telestes fontinalis*	C6	MG792941	0.004	0.008	0.005	0.015	0.009	0.016	0.004	0.013	0.003	0.003	0.003	0.003	0.006	0.004	0.003	0.003	0.003	0.003	0.003	0.003				
22	*Telestes karsticus*	C7	MG792943	0.004	0.008	0.005	0.015	0.009	0.016	0.004	0.013	0.003	0.003	0.003	0.003	0.006	0.004	0.003	0.003	0.003	0.003	0.003	0.003	—			
23	*Telestes metohiensis*	B13	MG792945	0.007	0.011	0.008	0.018	0.012	0.019	0.001	0.016	—	—	0.006	—	0.009	0.007	0.006	0.006	0.006	—	—	0.006	0.003	0.003		
24	*Telestes montenigrinus*	A10	MG792948	0.007	0.010	0.007	0.015	0.004	0.015	0.013	0.013	0.012	0.012	0.006	0.012	0.011	0.009	0.008	0.008	0.006	0.012	0.012	0.006	0.009	0.009	0.012	
25	*Telestes pleurobi-punctatus*	G7	MG792949	0.004	0.008	0.005	0.015	0.009	0.016	0.008	0.013	0.007	0.007	0.003	0.007	0.006	0.002	0.001	0.001	0.003	0.007	0.007	0.003	0.004	0.004	0.007	0.009

Distances are based on partial 18S rDNA combined with ITS1. Identical sequences are marked by dashes (—).

**Table 3:** Uncorrected pairwise genetic distances between individuals of *D. rarissimus* collected from different host species.

No.	Species	LocID	Accession number	1	2	3	4	5	6	7	8	9	10
1	*Alburnus neretvae*	B1	MG792844										
2	*Alburnus neretvae*	B2	MG792845	0.001									
3	*Pelasgus laconicus*	G11	MG792890	0.025	0.024								
4	*Rutilus basak*	B10	MG792895	0.020	0.019	0.020							
5	*Rutilus lacustris*	B13	MG792899	0.008	0.007	0.017	0.016						
6	*Rutilus ohridanus*	A4	MG792903	0.017	0.016	0.020	0.008	0.016					
7	*Rutilus rutilus*	CZ1	AJ564151	0.009	0.008	0.020	0.017	0.003	0.017				
8	*Telestes alfiensis*	G15	MG792938	0.030	0.029	0.025	0.025	0.022	0.027	0.025			
9	*Telestes dabar*	B12	MG792939	0.021	0.020	0.022	0.018	0.014	0.020	0.014	0.028		
10	*Telestes fontinalis*	C6	MG792940	0.022	0.021	0.024	0.022	0.017	0.020	0.014	0.028	0.010	
11	*Telestes metohiensis*	B13	MG792944	0.023	0.022	0.018	0.020	0.014	0.022	0.017	0.028	0.004	0.012

Distances are based on partial 18S rDNA combined with ITS1.

**Table 4:** Uncorrected pairwise genetic distances between individuals of *D. folkmanovae* collected from *Squalius* species.

No.	Species	LocID	Accession number	1	2	3	4	5	6	7	8
1	*Squalius cephalus*	B7	MG792911								
2	*Squalius cephalus*	CZ1	MG792912	0.002							
3	*Squalius orpheus*	G9	MG792916	0.018	0.020						
4	*Squalius platyceps*	A8	MG792919	0.016	0.018	0.017					
5	*Squalius prespensis*	A9	MG792921	0.011	0.013	0.013	0.009				
6	*Squalius prespensis*	G1	MG792922	0.010	0.012	0.011	0.007	0.002			
7	*Squalius* sp.	G10	MG792926	0.018	0.020	0.017	0.014	0.013	0.011		
8	*Squalius squalus*	C4	MG792928	0.035	0.037	0.035	0.032	0.028	0.026	0.036	
9	*Squalius vardarensis*	G4	MG792935	0.017	0.019	0.017	0.013	0.010	0.008	0.016	0.032

Distances are based on partial 18S rDNA combined with ITS1.

### Species delimitation

**Figure 3:**
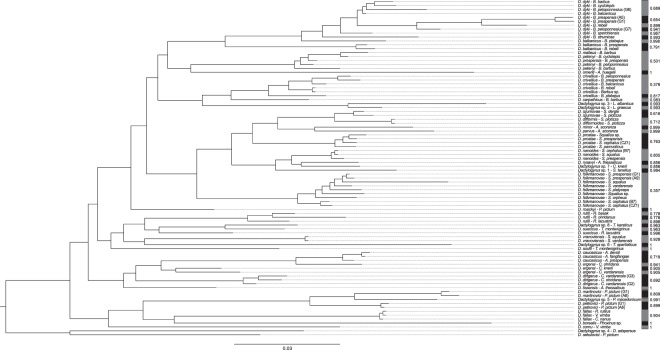
*Results of species PTP delimitation analysis based on the phylogram in* Fig. 2. Vertical bars at terminal branches indicate different species. Values along brackets indicate support values from both maximum likelihood partition and heuristic bayesian search. Species are the same as in Fig. 2 but several branches are rotated.

The species status of *Dactylogyrus* parasites exhibiting high interpopulation molecular diversity was investigated on the basis of a statistical analysis of our sequence data using PTP. We examined all specimens from clade 2 (Fig. 2). Results of the maximum likelihood analysis (Fig. 3) supported the original species statuses of specimens identified under the following species: *D. dirigerus, D. difformis, D. difformoides, D. izjumovae, D. nanoides, D. prostae, D. folkmanovae*, and *D. vranoviensis*. Specimens of *D. rutili*, collected from three *Rutilus* species, were recognized as three different species. Meanwhile, two molecular variants of *D. suecicus* and the phylogenetically closely related *Dactylogyrus* sp. 8 from *T. karsticus* were also recognized by our analyses as three different species. With respect to *D. dyki*, our analyses suggested six different species. *Dactylogyrus ergensi* specimens from *C. vardarensis, C. knerii*, and *S. squalus* were suggested to be three different species. *Dactylogyrus ergensi* from *C. ohridana* was suggested to be the same species as *D. caucasicus* from *Alburnoides*. Finally, *D. petenyi, D. prespensis* and *D. malleus* were identified as a single species on the basis of clustering methods. The strongest Bayesian supported solution was in congruence with the results of the maximum likelihood solution.

## Discussion

The present study suggests that the diversity of *Dactylogyrus* species parasitizing endemic cyprinids in the Balkans is poorer when compared to the diversity of *Dactylogyrus* from central European cyprinids and from cyprinids with a large distribution range (e.g. Šimková *et al*.^11^ documented up to 9 different *Dactylogyrus* species from widely distributed *Rutilus rutilus* in the Czech Republic). High numbers of *Dactylogyrus* species were also observed on African cyprinids from the genus *Labeo*, such as *L. coubie* with 9 *Dactylogyrus* species^46^. In contrast, we observed a maximum of 5 *Dactylogyrus* species on a single cyprinid species. These numbers are consistent with previous observations of southern European *Dactylogyrus* fauna, where no more than 5 species were collected from one cyprinid host species^7,44,45^. Such low *Dactylogyrus* species diversity probably has several causes. The distribution range of host species highly influences parasite diversity^47^. Our observations support Gregory’s hypothesis^37^, i.e. fish species with a wide distribution range are exposed to more parasite species; therefore, they exhibit high parasite diversity. Another potential explanation could be the following: host species with a wide distribution range include a much higher number of populations in comparison to endemic species, which favours parasite speciation. This is illustrated in the present study by *R. rutilus* and *R. aula*. While *R. rutilus*, referred to above as a species with a high *Dactylogyrus* species richness, is the cyprinid species with the widest distribution range in Europe, the distribution area of *R. aula* is limited to the Adriatic basin in Italy and the northwestern Balkans (the Northern Adriatic ichthyogeographical district^15^). *R. aula* is parasitized by a single *Dactylogyrus* species – namely, *D. erhardovae –* in contrast to the aforementioned *R. rutilus*^11^. A similar example concerns the Balkan endemic species *S. illyricus* or *S. peloponensis*, which exhibit very low *Dactylogyrus* species richness (i.e. single species) in comparison to *Squalius cephalus*, from which Seifertová *et al*.^38^ documented 9 different *Dactylogyrus* species (up to 14 *Dactylogyrus* species according to the checklist by Moravec^8^). Time of the year when the sampling is performed and the number of investigated populations are known to impact parasite diversity^47,48^. Data on *Dactylogyrus* diversity in cyprinids in central Europe are compiled from numerous studies (i.e. the checklist compiled by Moravec^8^) and include several sampling periods from different river basins, while the present study is focused on a single sampling period in a specific region. The investigated cyprinid hosts endemic to the Balkans are generally distributed in a restricted region where the number of populations potentially harbouring different parasites is expected to be rather lower than in central Europe. Therefore, also following Gregory’s hypothesis, we expected lower parasite diversity in endemic cyprinids with a restricted distribution range. Only a few host species, such as *S. squalus*, were collected from several distinct localities; however, the different host populations did not differ in their numbers of *Dactylogyrus* species. It was also shown that the composition of monogenean communities is influenced by environmental factors, especially water temperature. In such cases, shifts in the species compositions of monogenean communities within host species were observed throughout the year^49–53^.

The present phylogenetic analyses revealed four well-to-moderately supported clades including both endemic and non-endemic *Dactylogyrus* species, while four species – namely, *D. erhardovae, D. crucifer, D. caballeroi*, and *D. rarissimus* (all parasites of *Rutilus* spp.) – had external positions to these clades. *Dactylogyrus erhardovae* is considered to be a genus specific parasite of *Rutilus*, the first description of this species originating from *R. rubilio*^54^, an endemic species of the Apennine Peninsula^55,56^. In the Balkans, *Dactylogyrus erhardovae* was also found on *R. aula* and *R. basak*, phylogenetically closely related species^26,57^ distributed in the rivers of the Adriatic Sea basin, which is the proximal ichthyogeographic district to the Tyrrhenian Sea basin, where *R. rubilio* occurs. *Dactylogyrus crucifer* was originally described from *Rutilus rutilus*, but Šimková *et al*.^12^ collected this species also from *Leuciscus idus* and *Scardinius erythrophthalmus* and therefore suggested that *D. crucifer* represents a generalist species. In our study, *D. crucifer* was only collected from *Rutilus* species (*R. rutilus* from the Czech Republic and *R. lacustris* from the Ponto-Caspian area), which supports the association between *Rutilus* hosts and *D. crucifer* and even indicates that the occurrence of this parasite on other cyprinid species may be the result of accidental infection. Both *Rutilus* species parasitized by *D. crucifer* originated and live in sympatry in the Black Sea and Caspian Sea basins^58^, which may promote the host switching of *D. crucifer* between these two sister *Rutilus* lineages.

Interestingly, we showed that *Dactylogyrus* sp. 4 from *D. adspersus* and *D. sekulovici* from *P. pictum* clustered together (group 1). Both *Dactylogyrus* species seem to be host specific - at least, there are no previous records of these two species from other cyprinid species. Regarding the morphology of the hard parts, these two *Dactylogyrus* species differ in the shape of their MCOs. While *Dactylogyrus* sp. 4 has hard parts morphologically similar to those of *D. erhardovae* from *Rutilus*, it shares with *D. sekulovici* only the shape of the haptoral connective bars (see Pugachev *et al*.^45^ for morphology of *D. sekulovici*). Two cyprinid species – namely, *D. adspersus* and *P. pictum* – are representatives of two phylogenetically unrelated ancient lineages^26^, but have a similar geographical distribution, i.e. they are restricted to the rivers of the Adriatic Sea Basin. *Pachychilon pictum* occurs only in the Albanian ichthyogeographical district^59^; *D. adspersus* inhabits the central Adriatic (Dalmatian) district, which shares only two species with the Danubian basin^59–61^, and is probably linked to the Adriatic district by underground connections^16^. The paraphyly of the *Dactylogyrus* species from *P. pictum* suggests their multiple origin on this host. The phylogenetic proximity of *D. sekulovici* to *Dactylogyrus* sp. 4 suggests a host switch between two cyprinid species living in the same area of the central Adriatic region. The second host-specific parasite of *P. pictum* is *D. ivanovichi*^44,45^. Its phylogenetic position suggests a different origin (when compared to *D. sekulovici*), likely also resulting from a host switch. *Dactylogyrus ivanovichi* is phylogenetically closely related to *D. auriculatus* from *Abramis brama*. The two species exhibit MCOs with an identical structure and differ only in the positioning of the VA and in the root lengths of haptoral anchor hooks^45^. These two species, like the two species of the sister clade (clade 3), secondarily lost their connective haptoral ventral bar^45^. The phylogenetic proximity of *D. ivanovichi* and *D. auriculatus* and the morphological similarities in copulatory organs between *D. ivanovichi* and *Dactylogyrus* spp. of *A. brama* suggest that *D. ivanovichi* originated from a recent host switch from the widely distributed *A. brama*, and then adapted its attachment organ to new host species. Other *Dactylogyrus* species from *P. pictum*, namely *D. martinovici* and *D. petkovici*, are phylogenetically closely related to *Dactylogyrus* sp. 5 of *P. macedonicum*. *Dactylogyrus martinovici*, *D. petkovici*, and *Dactylogyrus* sp. 5 exhibit haptoral hard parts with an almost identical shape but differ in the shapes of their copulatory organs. This is in congruence with Šimková *et al*.^6^, suggesting similar adaptations of the haptor among *Dactylogyrus* species parasitizing phylogenetically related hosts. We can hypothesize that these three species evolving from the same ancestor have for a long time been associated with *Pachychilon* and that *D. martinovici* and *D. petkovici* emerged as a result of more recent intra-host duplication followed by reproductive isolation. In contrast, *D. ivanovichi* and *D. sekulovici* are the result of earlier host switching between cyprinid species of different genera living in contact zones and of subsequent speciation. Finally, another *Dactylogyrus* species from *P. pictum*, *D. rosickyi*, exhibits a different phylogenetic position when compared to the aforementioned *Dactylogyrus* of *Pachychilon* spp., which suggests a different origin for this species.

Regarding *Dactylogyrus* from *Barbus* spp., our analyses did not fully resolve the phylogenetic relationships between these species, but in general all species are clustered in three well or moderately supported groups (G–I). In total, we collected 5 different *Dactylogyrus* species from 10 *Barbus* hosts. The most common was *D. dyki*, parasitizing 8 *Barbus* species and representing one clade in our phylogenetic analysis. Šimková *et al*.^43^ observed significant interpopulational phenotypic plasticity and molecular variability among *D. dyki* isolated from 3 *Barbus* species, which is in accordance with the present study. The monophyly of the group including *D. dyki* specimens was supported. However, low support for *D. dyki* from *B. strumicae* was found and these specimens were recognized as a different species by species delimitation analysis. Following the suggestion of Šimková *et al*.^43^, *D. dyki* from *Barbus* spp. could represent a species complex of several morphologically similar species. The confirmation of this hypothesis requires further morphological reevaluation of *Dactylogyrus* representatives from all *Barbus* hosts, including those from *B. meridionalis* in Western Europe and *B. tyberinus* from the Apennines. We inferred some paraphyly concerning *D. balkanicus*. Whilst *Dactylogyrus* specimens of *B. prespensis* and *B. rebeli* were clustered together, specimens from *B. plebejus* appeared to be phylogenetically related to *D. dyki*. The sister status of these two species is supported by the similar shape of the sclerotized parts of their haptors (both species share a small triangular connective ventral bar), and also the remarkably similar shape of their MCOs^45^. Both species were collected from *B. rebeli* and *B. prespensis*, phylogenetically closely related *Barbus* species^25,62^, suggesting (1) historical intra-host speciation, i.e. parasite duplication on their common ancestor and a later host switch to another endemic *Barbus*, or (2) parasite duplication on recent *Barbus* species in this region and a host switch to the phylogenetically and geographically closest *Barbus* species. According to our phylogenetic analyses, *D. petenyi*, *D. malleus*, and *D. prespensis* form a well-supported group, namely group G. These three *Dactylogyrus* species parasitizing *Barbus* species share similar morphologies of the copulatory organs and haptoral hard parts. Surprisingly, specimens of *D. petenyi* do not form a monophyletic group. Species delimitation analysis suggests that each representative of group G represents a single species.

Specimens of *D. crivellius* from different host species formed a monophyletic group. Our phylogenetic analyses support a monophyletic group including *D. crivellius* from Balkan *Barbus* spp., *D. carpathicus* from *B. barbus*, and *Dactylogyrus* sp. 2 and *Dactylogyrus* sp. 3. These four species exhibit the same morphology of a ventral bar with 5 extremities, a typical feature of *Dactylogyrus* spp. from *Luciobarbus*. Species with this morphology are considered as the ‘carpathicus’^42^ or ‘cornu’^45^ type. This supports the hypothesis that haptoral hard parts are more suitable for resolving the phylogeny of monogeneans; that is, haptor morphology is similar between closely related species^6,63,64^.

The phylogenetic position of *D. omenti* among *Dactylogyrus* species parasitizing *Barbus* and *Luciobarbus* was already suggested by Benovics *et al*.^65^. Even though its exact phylogenetic position is not fully resolved, our result suggests that this species is phylogenetically closer to *D. petenyi* and *D. prespensis* than to the aforementioned species which share the ‘cornu’ type of haptoral ventral bar. Adding more *Dactylogyrus* species from Iberian, North African, and Middle Eastern *Barbus* and *Luciobarbus* in a phylogenetic reconstruction and assessing coevolutionary scenarios involving these parasites and their hosts could better resolve the relationships within this group of *Dactylogyrus*.

Several well-supported phylogenetic groups (J–L) were formed exclusively by *Dactylogyrus* species of the ‘ergensi’ type of copulatory organ, or, in the case of *D. tissensis*, the ‘chondrostomi’ type of copulatory organ^47^. While the MCO and VA among *Dactylogyrus* spp. belonging to groups J–L are very similar, these species differ in the shapes and sizes of their haptoral hard parts. All *Dactylogyrus* species of groups K and L parasitize species of the genera *Alburnoides* and *Chondrostoma*. The species status of *D. caucasicus* parasitizing *Alburnoides* and that of *D. dirigerus* parasitizing *Chondrostoma* were supported by species delimitation analysis. Surprisingly, *Rutilus*-specific *D. rutili* belonging to the phylogenetically distant group J possesses the same type of copulatory organ as *D. caucasicus* and *D. dirigerus*. This suggests that a similar copulatory organ morphotype can emerge independently several times during the evolution of *Dactylogyrus* species in evolutionarily distant hosts (such are *Rutilus, Chondrostoma*, and *Alburnoides*^26^). Rohde^2^ hypothesized that the rapid evolution of morphological variation in copulatory organs is considered as a mechanism for avoiding hybridization. In contrast, similar types of copulatory organs in *Dactylogyrus* species may be recognized in different host lineages, as shown in the present study. Then, species with a similar MCO morphotype could be found within congeneric hosts only if these parasite lineages had diversified recently (e.g. *D. ergensi* and *D. dirigerus* of *Chondrostoma*).

High numbers of southern European endemic *Dactylogyrus* species were strictly host specific and/or distributed only in one region. However, some of them were collected from a wide range of cyprinid hosts. *Dactylogyrus vistulae* is the species with the widest host range in the Balkans. In addition to the host range for this parasite revealed in this study, the presence of *D. vistulae* was also reported from *R. rutilus* in Finland^66^ and from *V. vimba* in the Czech Republic^8^. Genetic distances between specimens collected from different host species correlated with geographical distances, suggesting the geographical structure of *D. vistulae* populations, rather than some association with the phylogenetic relatedness of the host species. For example, *D. vistulae* from *C. phoxinus* appears to be genetically more similar to *D. vistulae* from hosts in the same or close ichthyogeographical region than to *D. vistulae* collected from geographically separated congeneric *Chondrostoma*. Since *D. vistulae* is widely distributed and relatively easily distinguishable from other *Dactylogyrus* spp. on the same hosts (on the basis of morphological characters and its large body size^45^), it could potentially represent a suitable model for population studies that could elucidate the origin of this species and the distribution pattern between phylogenetically distant hosts or between two host species from different regions. Another species with a wide distribution range is *D. rarissimus*. It was originally considered as a specialist of *R. rutilus*^6,12,67^; however, we collected this species in the Balkans from phylogenetically well-separated genera: *Rutilus*, *Alburnus, Pelasgus* and *Telestes*. In this case, the Mantel test did not reveal a significant correlation between genetic and geographical distances, even as specimens collected from *T. alfiensis* and *P. laconicus* in Peloponnese (the only representatives of *D. rarissimus* from the Ionian ichthyogeographic district) are genetically the most different from northern populations originating from the Albanian district (such as *R. ohridanus*). We measured only a very small genetic difference between *D. rarissimus* from *R. rutilus* and *D. rarissimus* from *R. lacustris* (similarly to that measured for *D. crucifer*), which supports the recent divergence of these *Rutilus* species or, alternatively, a more ancient separation followed by recent contact. All these results suggest that *D. rarissimus* is a true generalist species parasitizing several cyprinid genera. We investigated the correlation between genetic and geographical distances among *D. folkmanovae* individuals. In contrast to *D. vistulae* and *D. rarissimus*, *D. folkmanovae* was reported as a generalist parasite of *S. cephalus* and *R. rutilus*^8,67^; however, it is generally reported in *Squalius* species^12^ and, in the Balkans, *D. folkmanovae* occurs strictly on *Squalius* spp. *Dactylogyrus folkmanovae* from *S. squalus* appeared to be the most genetically different from individuals parasitizing other host species. Of the southern European endemic *Squalius* species, *Squalius squalus* exhibits the largest distribution range, i.e. it covers the whole peri-Adriatic region^15^, and is phylogenetically closely related to *S. prespensis*^26^. This is in congruence with measurements of genetic distance, according to which *D. folkmanovae* of *S. squalus* and *S. prespensis* are the most similar. These results suggest that *D. folkmanovae* of *S. squalus* is the oldest lineage within this species in the Balkans. In contrast, representatives of *D. folkmanovae* from *S. cephalus* in the Czech Republic and *D. folkmanovae* from *S. cephalus* in Bosnia and Herzegovina are genetically very similar. These small genetic distances (in the case of both *D. vistulae* and *D. folkmanovae*) could be the result of more recent contact between hosts from these two distant regions via underground connections, as proposed by Palandačić *et al*.^16^, or through the introduction of non-native species/populations into the Balkan region. Fish introduction has been a very common occurence in the Balkans and includes both exotic, and native species from geographically near localities^68,69^. River drainages^70,71^ and also isolated karstic drainages are affected, where non-native species such as *S. cephalus* and *R. rutilus* have been introduced^72^. Low molecular variability between Czech and Bosnian-Herzegovinian populations of *D. folkmanovae* may favour the hypothesis of the natural dispersion of the fish via river connections. However, the investigation of other European populations and the use of other genetic markers suitable for population genetics of *Dactylogyrus* are necessary to reveal the distribution patterns of widespread *Dactylogyrus* species. In addition, the extent of parasite transfer from introduced species to endemic species needs to be studied further to reduce the possible risk of parasite introduction to already threatened native species.

In this study, we revealed interpopulation genetic variability within endemic Balkan *Dactylogyrus* species. The intraspecific genetic distances could also be linked to the morphological variability which was suggested for other monogenean taxa^73–75^. Concerning *Dactylogyrus*, morphological variability among the haptoral hard parts of a given *Dactylogyrus* species was recorded even within a single host specimen of *L. maghrebensis*^71^, but without any molecular variability, suggesting phenotypic plasticity and/or selection within a specific microhabitat. On the other hand, as documented above, our molecular data also revealed potential complexes of cryptic species, formerly considered to be a single species solely on the basis of a morphological approach. According to species delimitation analysis, the 38 *Dactylogyrus* species included in the analysis may in fact represent 47 species. This finding is in accordance with previous studies, in which delimitation analyses were incongruent with classical taxonomy^76,77^. In our study, *Dactylogyrus* sp. 2 and *Dactylogyrus* sp. 3 from *L. graecus* and *L. albanicus*, respectively, were shown to be morphologically indistinguishable species; however, molecular data suggest that they are actually two different species (which is also supported by species delimitation analysis). A similar result was revealed for other *Dactylogyrus* species, such as *D. rutili*, which seems, on the basis of delimitation analysis, to represent three species parasitizing three host species, and *D. dyki*, which seems to represent six potential species on 10 *Barbus* host species. Our future aim will be to undertake the morphometrical reevaluation of taxonomically important traits in combination with the use of molecular data in order to resolve the potential species complexes previously recognized within *Dactylogyrus*^76^.

## Material and Methods

### Parasite sampling

**Figure 4:**
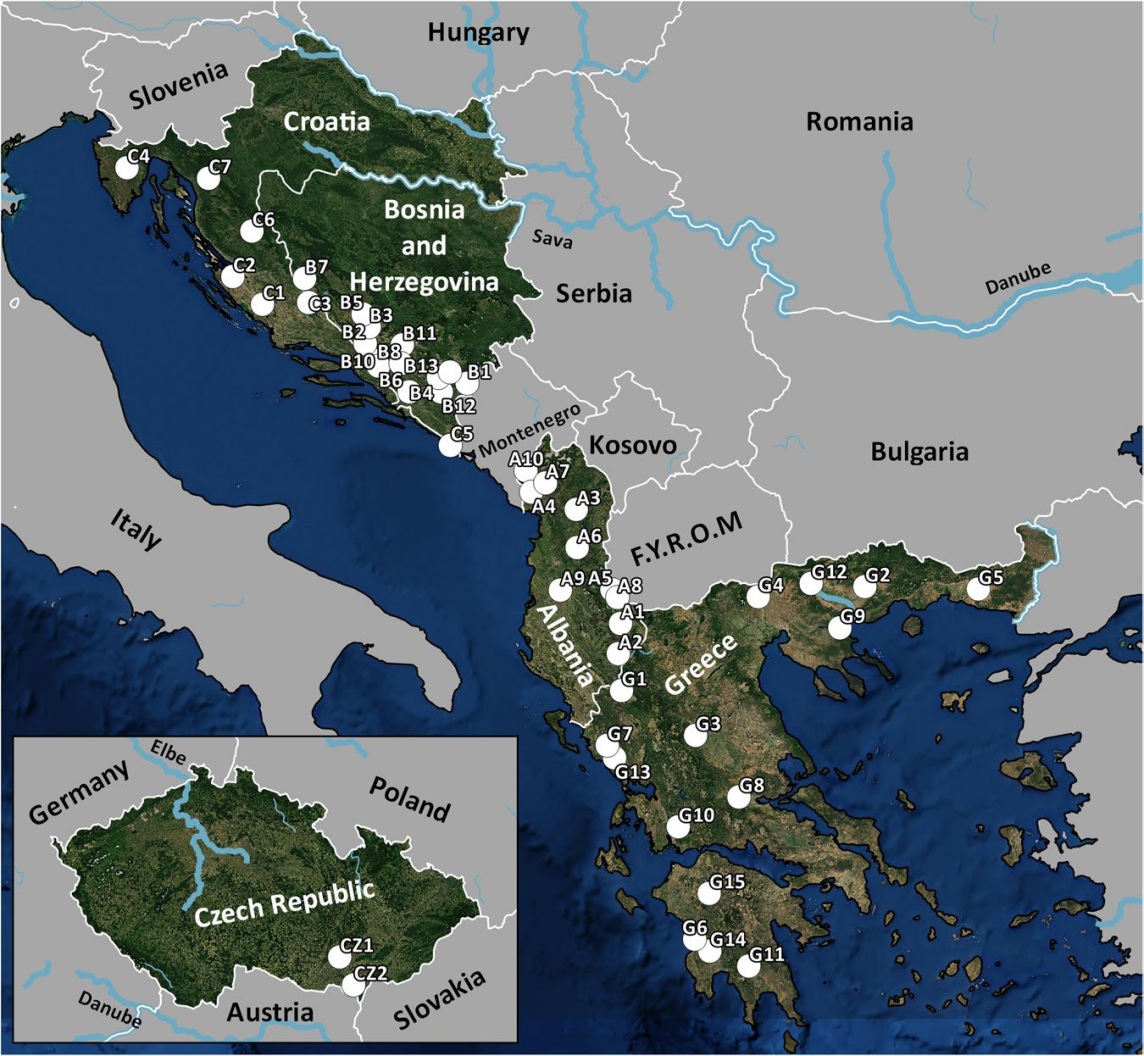
*Map of collection localities in the Balkans*. The sames codes for localities are used in tables under the label LocID. The map was generated in QGIS 3.0.3^94^.

From 2014 to 2017, individuals from 63 cyprinid fish species were sampled from 47 different localities in the Balkan Peninsula and the Czech Republic (Table 5, Fig. 4). Approximately 90% of all endemic cyprinid species in the Balkans were processed in this study^15^. Fish were dissected using the standard methods described by Ergens and Lom^78^ and their *Dactylogyrus* species were collected. More precisely, *Dactylogyrus* specimens were removed from the gills, mounted on slides, and covered in a mixture of glycerine and ammonium picrate (GAP^79^) for further determination. All applicable institutional, national and international guidelines for the care and use of animals were followed and approved by the Animal Care and Use Committee of the Faculty of Science, Masaryk University in Brno (Czech Republic). Identification at the species level was performed using an Olympus BX51 microscope equipped with phase contrast optics. *Dactylogyrus* species were determined using Pugachev *et al*.^45^ on the basis of the size and shape of the hard parts of the attachment organ (the haptor) and the reproductive organs (MCO and VA). Some *Dactylogyrus* specimens from each cyprinid species investigated were bisected using fine needles under a dissecting microscope, and the body part with the haptor was individually preserved in 96% ethanol for further DNA extraction. The remaining body part, i.e. that including the hard parts of the respective reproductive organ, was mounted on a slide for species determination.

**Table 5:** List of cyprinid species including the localities of their collection.

Host	LocID	NH	N	Locality	Main river basin	Coordinates
*Abramis brama*	CZ1	5	2	Svratka River	Danube	49°05′32.01″N 16°37′11.00″E
*Alburnoides devolli*	A1	6	1	Devoli, Maliq	Seman	40°42′57.07″N 20°40′54.06″E
*Alburnoides fangfangae*	A2	7	1	Osum, Vodice	Seman	40°24′13.07″N 20°39′04.04″E
*Alburnoides ohridanus*	A3	10	1	Fani i Vogel, Reps	Seman	41°52′51.01″N 20°04′44.04″E
*Alburnoides prespensis*	G1	5	1	Aoos, Kalithea	Aoos	40°01′16.67″N 20°41′40.19″E
*Alburnoides strymonicus*	G2	5	2	Angistis, between Alistrati & Drama	Strymon	41°05′42.08″N 24°00′18.29″E
*Alburnoides thessalicus*	G3	12	3	Pinios, Rongia - Valamandrio	Pinios	39°33′07.85″N 21°42′08.02″E
*Alburnus neretvae*	B1	7	2	Mušnica, Avtovac	Neretva	43°08′42.05″N 18°35′45.00″E
	B2	10	2	Zagorje, Jabuke	Neretva	43°32′18.53″N 17°12′34.28″E
*Alburnus scoranza*	A4	5	2	Skadar lake, Shiroke	Ohrid-Drin-Skadar lake system	42°03′24.94″N 19°28′07.05″E
*Aulopyge hugelii*	B3	14	2	Šujica, Duvansko Polje	Neretva	43°42′05.07″N 17°15′50.05″E
*Barbus balcanicus*	G4	5	3	Gallikos, Mandres	Gallikos	40°59′28.35″N 22°33′14.49″E
*Barbus barbus*	CZ1	5	3	Svratka River	Danube	49°05′32.01″N 16°37′11.00″E
*Barbus cyclolepis*	G5	3	2	Macropotamos River	Filiouri	41°04′13.00″N 25°32′52.00″E
*Barbus peloponnesius*	G6	8	1	Neda, Gianitsochori	Neda	37°23′04.34″N 21°41′24.15″E
	G7	5	3	Kokitos, Pagrati	Acheron	39°26′53.02″N 20°30′03.06″E
*Barbus plebejus*	C1	7	2	Bribirske Mostine, Bribišnica	Krka	43°55′28.21″N 15°48′45.07″E
*Barbus prespensis*	A5	5	1	Shkumbini, Perrenjas	Shkumbini	41°03′50.09″N 20°33′56.06″E
	G1	5	4	Aoos, Kalithea	Aoos	40°01′16.67″N 20°41′40.19″E
*Barbus rebeli*	A6	7	3	Mat, Klos	Mat	41°29′37.01″N 20°05′29.04″E
*Barbus* sp.	A7	6	1	Kiri	Ohrid-Drin-Skadar lake system	42°08′56.02″N 19°39′42.01″E
*Barbus sperchiensis*	G8	4	1	Sperchios, Ypati	Sperchios	38°54′14.33″N 22°17′30.22″E
*Barbus strumicae*	G9	5	1	Rihios river, Stavros	Volvi lake	40°40′16.34″N 23°39′50.87″E
*Carassius gibelio*	CZ2	5	1	Dyje River	Danube	48°48′09.04″N 16°50′19.03″E
	C2	10	2	Baštica reservoir	Baštica	44°11′42.37″N 15°24′32.13″E
*Chondrostoma knerii*	B4	5	2	Rečina river, near Jelim lake, Hutovo Blato	Neretva	43°03′39.72″N 17°48′29.30″E
*Chondrostoma nasus*	CZ1	5	1	Svratka River	Danube	49°05′32.01″N 16°37′11.00″E
*Chondrostoma ohridana*	G1	4	3	Aoos, Kalithea	Aoos	40°01′16.67″N 20°41′40.19″E
*Chondrostoma phoxinus*	B5	11	1	Šujica, Šujicko Polje	Neretva	43°49′41.43″N 17°10′48.20″E
*Chondrostoma vardarensis*	G2	3	1	Angistis river, Koninogia	Strymon	41°11′36.41″N 23°54′25.00″E
	G2	2	1	Angistis, between Alistrati & Drama	Strymon	41°05′42.08″N 24°00′18.29″E
	G3	1	2	Pinios, Rongia - Valamandrio	Pinios	39°33′07.85″N 21°42′08.02″E
*Delminichthys adspersus*	B6	6	1	Nezdravica, Tihaljina	Neretva	43°19′00.05″N 17°23′20.01″E
*Luciobarbus albanicus*	G10	4	1	Trichonis lake, Panetolio	Acheloos	38°35′20.19″N 21°28′02.68″E
*Luciobarbus graecus*	G7	10	1	Sperchios, Ypati	Sperchios	38°54′14.33″N 22°17′30.22″E
*Pachychilon macedonicum*	G3	8	1	Pinios, Rongia - Valamandrio	Pinios	39°33′07.85″N 21°42′08.02″E
*Pachychilon pictum*	A8	4	2	Ohrid lake	Ohrid-Drin-Skadar lake system	41°04′27.08″N 20°37′40.00″E
	G1	5	5	Aoos, Kalithea	Aoos	40°01′16.67″N 20°41′40.19″E
*Pelasgus laconicus*	G11	13	1	Evrotas, Sparti	Evrotas	37°05′34.70″N 22°25′34.81″E
*Phoxinellus alepidotus*	B7	12	1	Bosansko Grahovo, Korana river	Korana	44°10′37.00″N 16°23′03.61″E
*Phoxinellus pseudalepidotus*	B8	10	1	Lištica, Polog	Neretva	43°20′32.09″N 17°41′37.04″E
*Phoxinus* sp.	B9	14	1	Zalomka, Ribari	Neretva	43°15′26.04″N 18°21′41.05″E
*Rutilus aula*	C2	10	1	Baštica river, Grabovač reservoir	Baštica	44°11′42.37″N 15°24′32.13″E
*Rutilus basak*	B10	13	4	Krenica lake, Drinovci	Neretva	43°22′25.00″N 17°19′59.04″E
*Rutilus lacustris*	G12	3	4	flood pools by Struma, Lithopos	Strymon	41°07′40.41″N 23°16′24.70″E
*Rutilus ohridanus*	A4	4	4	Skadar lake, Shiroke	Ohrid-Drin-Skadar lake system	42°03′24.94″N 19°28′07.05″E
*Rutilus rutilus*	CZ1	5	3	Svratka River	Danube	49°05′32.01″N 16°37′11.00″E
*Scardinius dergle*	C1	10	1	Bribirske Mostine, Bribišnica	Krka	43°55′28.21″N 15°48′45.07″E
*Scardinius plotizza*	B4	7	3	Rečina river, near Jelim lake, Hutovo Blato	Neretva	43°03′39.72″N 17°48′29.30″E
*Squalius cephalus*	CZ1	5	2	Svratka River	Danube	49°05′32.01″N 16°37′11.00″E
	B7	4	2	Bosansko Grahovo, Korana river	Korana	44°10′37.00″N 16°23′03.61″E
*Squalius illyricus*	C3	8	1	Cetina river, Kosore	Cetina	43°56′29.78″N 16°26′23.37″E
*Squalius orpheus*	G9	4	1	Rihios river, Stavros	Volvi lake	40°40′16.34″N 23°39′50.87″E
*Squalius pamvoticus*	G13	6	1	Acheron, Gliki	Acheron	39°19′00.05″N 20°36′04.03″E
*Squalius peloponensis*	G14	5	1	Pamissos, Vasiliko	Pamissos	37°15′17.39″N 21°53′45.15″E
*Squalius platyceps*	A8	5	2	Ohrid lake	Ohrid-Drin-Skadar lake system	40°59′00.66″N 20°38′23.40″E
*Squalius prespensis*	A9	4	2	Shkumbini, Pajove	Shkumbini	41°03′31.07″N 19°51′47.03″E
	G1	6	3	Aoos, Kalithea	Aoos	40°01′16.67″N 20°41′40.19″E
*Squalius* sp.	G10	2	2	Trichonis lake, Panetolio	Acheloos	38°35′20.19″N 21°28′02.68″E
*Squalius squalus*	B11	10	3	Donja Drežnica, Drežnica river	Drežnica	43°31′31.46″N 17°42′51.66″E
	C4	11	1	Pazin, Pazinčica river	Pazinčica	45°14′47.92″N 13°58′10.66″E
*Squalius svallize*	C5	15	1	Konavočica, Grude	Ljuta	42°31′33.86″N 18°22′04.16″E
*Squalius tenellus*	B5	11	2	Šujica, Šujičko Polje	Neretva	43°49′41.43″N 17°10′48.20″E
*Squalius vardarensis*	G4	4	3	Gallikos, Mandres	Gallikos	40°52′07.33″N 22°53′59.12″E
*Telestes alfiensis*	G15	5	1	Erimantos, Tripotamo	Alfios	37°52′37.07″N 21°53′15.05″E
*Telestes dabar*	B12	3	1	Vrijeka, Dabarsko Polje	Neretva	43°03′32.07″N 18°14′39.04″E
*Telestes fontinalis*	C6	13	2	Krbavsko polje, Laudonov gaj	Krbava	44°38′14.33″N 15°40′05.65″E
*Telestes karsticus*	C7	10	2	Drežnica, Sušik river	Drežnica	45°08′44.13″N 15°04′41.56″E
*Telestes metohiensis*	B13	5	2	Zalomka, Nevesinjsko polje	Neretva	43°12′06.06″N 18°12′21.07″E
*Telestes montenigrinus*	A10	10	3	Skadar lake, Shegan	Ohrid-Drin-Skadar lake system	42°16′22.09″N 19°23′39.09″E
*Telestes pleurobipunctatus*	G7	6	1	Kokitos, Pagrati	Acheron	39°26′53.02″N 20°30′03.06″E
*Tropidophoxinellus spartiaticus*	G6	5	1	Neda, Gianitsochori	Neda	37°23′04.34″N 21°41′24.15″E
*Vimba vimba*	CZ1	5	3	Svratka River	Danube	49°05′32.01″N 16°37'11.00″E

LocID = codes used in all tables and figures, NH = number of host specimens processed, N = number of *Dactylogyrus* species collected.

### DNA extraction, amplification, and sequencing

Individual parasites were removed from the ethanol and dried using a vacuum centrifuge. DNA was extracted using the standard protocol (DNeasy Blood & Tissue Kit, Qiagen, Hilden, Germany). Partial 18S rDNA and the the entire ITS1 region were amplified using the primers S1 (5′-ATTCCGATAACGAACGAGACT-3′) and IR8 (5′-GCTAGCTGCGTTCTTCATCGA-3′)^80^, which anneal to the 18S and 5.8S rDNA respectively. Partial 28S rDNA was amplified using the following primers: forward C1 (5′-ACCCGCTGAATTTAAGCA-3′) and reverse D2 (5′-TGGTCCGTGTTTCAAGAC-3′)^81^. Each amplification reaction for partial 18S rDNA and the ITS1 region was performed in a final volume of 15 µl, containing 1.5 units of Taq polymerase, 1X buffer, 1.5 mM MgCl_2_, 0.2 mM of each dNTP, 0.5 µM of each primer, and 2.5 µl of DNA (20 ng/µl). PCR was carried out using the following steps: 2 min at 94 °C, followed by 40 cycles of 1 min at 94 °C, 1 min at 53 °C, and 1 min 30s at 72 °C, and 10 minutes of final elongation at 72°C. The PCR for partial 28S was performed using the same conditions as described in Šimková *et al*.^82^. The PCR products were checked on 1% agarose gel and purified using ExoSAP-IT kit (Ecoli, Bratislava, SK) following the standard protocol. Purified products were directly sequenced using the PCR primers and BigDye Terminator Cycle Sequencing kit (Applied Biosystems, Foster City, CA). Sequencing was performed on an ABI 3130 Genetic Analyzer (Applied Biosystems). New sequences were deposited in GenBank (their accession numbers are shown with asterisks in Table 5).

### Phylogenetic analyses

DNA sequences were aligned using fast Fourier transform in MAFFT^83^. The sequences were trimmed to concur with *Dactylogyrus* sequences obtained from GenBank. The sequences for 14 *Dactylogyrus* species from central European cyprinids were obtained by sequencing in this study or acquired from GenBank (see Table 5 for accession numbers).

Genetic distances between specimens of selected *Dactylogyrus* species collected from different host species were computed using sequences of partial 18S rDNA combined with ITS1 region. Uncorrected pairwise distances were calculated in MEGA 7^84^.

Gaps and ambiguously aligned regions were removed from the alignment using GBlocks v. 0.91^85^. Phylogenetic analyses using maximum likelihood were computed with RaxML v8.1.X^86^, and by means of Bayesian inference with MrBayes 3.2^87^. For each analysis, jModelTest 2.1.10 was employed to select the most appropriate model of DNA evolution^88,89^ using the Bayesian information criterion (BIC). Trees obtained by ML analyses were validated using 1000 bootstrap iterations. Bayesian inference was performed using the Metropolis-coupled Markov chain Monte Carlo algorithm, with 2 parallel runs of 1 cold and 3 hot chains. This was run for 10^7^ generations and trees were sampled every 10^2^ generations. 30% of all saved trees were discarded as a relative burn-in period according to the standard deviation split frequency value (<0.01).

Phylogenetic reconstruction including all sampled *Dactylogyrus* species was based on concatenated sequences of partial 18S rDNA and partial 28S rDNA (Fig. 1). The resulting phylogram was rooted using the evolutionarily divergent lineage of *Dactylogyrus* species parasitising *Carassius gibelio* and *Cyprinus carpio*^12^. To resolve the phylogenetic relationships among specific subgroups, partial subtree analyses were performed using partial 18S rDNA combined with the ITS1 region and partial 28S rDNA. Optimal evolutionary models were selected for each marker using BIC, each model including an alpha parameter for the gamma distribution (G) accounting for rate heterogeneity across sites and/or a proportion of invariable sites (I).

Species delineation in the final trees was carried out using a PTP (Poisson Tree Processes) model^90^. This approach was applied to the BI tree computed from concatenated partial 18S rDNA, 28S rDNA, and the partial ITS1 region, and run for 5 × 10^5^ generations. 30% of the resulting trees were discarded as burn-in. PTP can give species delimitation hypothesis based on gene trees inferred from molecular sequences, modelling the speciation or branching events in terms of the number of mutations. This method does not require an ultrametric input tree or a sequence similarity threshold as input, but uses only the tree resulting from either phylogenetic reconstruction.

The Mantel test^91^ to test the correlation between genetic and geographical distances was performed in R^92^ using the *mantel* function in the *vegan* package^93^.

## Data Availability

All new sequences of *Dactylogyrus* obtained during this study were deposited in NCBI GenBank under accession numbers MG792838–MG793066. Appropriate accession numbers according to *Dactylogyrus* species and specific rDNA regions are presented in Tables 1–3. Since whole fish specimens were completely processed during parasitological dissection, additional specimens of each analysed host species were collected from the same locality and fish vouchers were deposited in the ichthyological collection of the National Museum in Prague (Czech Republic). Voucher specimens of the sequenced *Dactylogyrus* species (excluding undescribed species) are deposited in the Finnish Museum of Natural History in Helsinki (available under the accession numbers MZH KN10850–989).
